# Investigating attention toward pain-related cues in an Arabic-speaking population with and without chronic pain

**DOI:** 10.1007/s00221-024-06789-9

**Published:** 2024-02-29

**Authors:** Ahmad N. Abudoush, Ellen Poliakoff, Maria Panagioti, Alexander Hodkinson, Nusrat Husain

**Affiliations:** 1 Department of Psychology, Faculty of Arts, School of Arts, The University of Jordan, Amman, Jordan; 2 School of Health Sciences, Faculty of Biology Medicine and Health, The University of Manchester, Manchester, UK

**Keywords:** Attentional bias, Emotional posner spatial cueing task, Emotional stroop task, Inhibition of return, Chronic pain, Arabic population

## Abstract

There is some evidence for attentional biases in individuals with chronic pain (CP). Cultural and linguistic differences might affect the manifestation of these processes across populations. However, such attentional biases have not been explored in the Arabic-speaking population. The current study investigated these attentional biases and possible associations with resilience. Two matched groups of Arabic-speaking participants with (58) and without (58) CP were recruited from Jordan and the United Kingdom. They completed emotionally modified versions of the Posner cueing and Stroop tasks, alongside questionnaires. Significant group differences were found for the Posner task, with the CP group exhibiting disengagement revealed by the inhibition of return (IOR) effect for sensory pain-related cues compared to delayed disengagement for the other cue types. The control group showed IOR across cue types. No group differences were found on the Stroop task. The CP group had lower resilience scores than healthy controls, and resilience moderated performance on the Posner task. The study provides preliminary evidence about the attentional processes in the Arabic population; the speed of disengagement is affected in the CP group with early disengagement for sensory pain-related information compared to affect pain and neutral stimuli. Furthermore, resilience levels in the CP and control group moderated the performance on the Posner task, suggesting that it influences attentional allocation. This study can help in understanding how the phenomenon of attention bias intertwines with the cultural and linguistic factors. Future research should further explore attentional dynamics across different time points in this population and the modulatory effect of resilience.

**Supplementary Information:**

The online version contains supplementary material available at 10.1007/s00221-024-06789-9.

## Introduction

Chronic pain (CP) is pain that persists or reoccurs for three to six months or more despite treatment (Treede et al. 2015). Different theories have been developed to account for the psychological factors contributing to the development and maintenance of CP (Kuch 2001). Attention-CP-related models explain that attention toward and away from pain-related information might play a role in maintaining pain symptoms (Abudoush et al. 2023a, b). Previous meta-analyses confirmed that people with CP exhibit attentional biases related to pain-related information that differs with mild to moderate strength from those without (Crombez et al. 2013; Todd et al. 2018; Jones et al. 2021). However, despite recent advances in the field of CP-selective attention, such CP-selective attention-related experiments have not been adapted or replicated in the Arabic population to assess its cultural and linguistic appropriateness. Despite the scarcity of studies, the prevalence of CP in the Arabic population is reported to range between 20 and 46.4% (Elzahaf et al. 2016; Almalki et al. 2019). Further, previous studies conducted among different ethnic groups varied in their outcomes (Abudoush et al. 2023a, b), which might be attributed at least partially to the uniqueness of their cultural beliefs, practices, and experiences (Hedden et al. 2008). This view is also supported by neuroimaging studies that show cultural differences in the brain areas involved in attentional processes (Han and Northoff 2008). Thus, this study aimed to address the gap related to the Arabic population and integrate the evidence available from previous literature. Therefore, to expand our understanding of the relationship between attentional biases and CP, experimental tasks in this field need to be adapted and findings replicated in the Arabic population.

When an individual is exposed to threat-related information, several physiological responses take place in the body to protect, it as a survival mechanism (March et al. 2022). More than a half-century ago, the narrow attention phenomenon became an influential concept around scanning and focusing on objects, which linked with threat as a survival mechanism (Wachtel 1967). As the individual concentrates on the threat, the width of their visual-spatial range decreases, allowing the person to focus attention on the threat source and plan a suitable survival response. In relation to CP, different models of the CP-attention association and related processes have been proposed. These involve attending toward or away from pain-related information (Abudoush et al. 2023a, b) and may be modulated by the level of threat. For instance, the vigilance-avoidance hypothesis asserts that hypervigilance is linked with attending more strongly initially, when a threat appears for a short duration (< 500 ms), and then subsequent avoidance (> 500 ms) (Mogg et al. 2004). Derakshan et al. (2008) linked the pattern of initial vigilance, which is followed by avoidance, with having dysfunctional self-relevant schematic information. The threat interpretation model (Todd et al. 2015) asserts that individuals with CP exhibit hypervigilance toward pain-related information, which is positively correlated with the threat level at the early stage of attention. However, in the sustained attention model, low, and high levels of interpreted threat result in avoidance, while a moderate level results in a difficulty of disengagement of attention from the threat (Todd et al. 2015).

Socio-cultural factors can influence the performance on selective attention-related tasks (Caparos et al. 2013). These factors have been especially explored in young children (Jurkat et al. 2020). For instance, Senzaki et al. (2018) found differences in performance on selective attention-related tasks between Eastern and Western children depending on their socio-cultural context. Further, neural structure and functions are influenced by sustained cultural experiences (Park and Huang 2010). A recent study explored the neuroanatomical differences in the processing of the Arabic language compared to an Indo-European language (i.e., German) and found that different environmental factors (including cultural variations) influence the linguistic processing related to the brain structural language connectome (Wei et al. 2023). However, investigations of CP-attention have not explored culturally specific factors among the Arabic population. Thus, understanding these potential differences is essential among different populations. Some studies have taken place in different countries that brought up cultural context factors, such as meanings of terms used, yet there is no clear evidence about the effect of such factors on attentional bias processes (Mohammadi et al. 2012; Abudoush et al. 2023a, b). As there are no previous similar studies in the Arabic population, it is essential to understand these selective attention processes in the adult group before exploring them in other more vulnerable age groups (i.e., children, teenagers, and older adults).

Researchers have used different tasks to try to objectively measure attentional biases toward or away from a threatening stimulus (i.e., words or pictures) using reaction times in visual tasks including the cue-target task (Posner 1980), the Dot-probe task (MacLeod et al. 1986), and the emotional version of the Stroop task (Williams et al. 1996). A recent systematic review and meta-analysis has compared and summarized the results of studies that included these tasks in light of the theoretical frameworks and related attention processes (Abudoush et al. 2023a, b). In the Posner task, the individual’s ability to shift their attention between spatial locations is assessed; the spatial cue presented is either endogenous (i.e., informs the participant where the upcoming target is likely to appear) or exogenous (i.e., the cue presented at a possible target location but is non-predictive, that it does not indicate where the target will appear). This cue is followed by the target appearance, either in the same location to the stimulus (also called cued or valid trial), or in the opposite location to the stimulus (also called uncued or invalid trial). The Posner cueing task involves different spatial locations and the engagement (and possible subsequent disengagement) of attention with these cues. The Posner task can be used to measure faster orientation of attention toward the cue (facilitation) and faster (or slower) disengagement of attention from the cue. At later time points after cue presentation, inhibition of return (IOR) can be found where people are slower to reorient to the previously attended (cued) location (Klein 2000). To assess spatial attention toward threatening stimuli the Posner task is modified using the threatening stimulus (i.e., pain-related information) as a cue, that is presented in the same (i.e., valid cue) or opposite location (i.e., invalid cue) to the target. The Stroop task uses the congruency and incongruency between the word meaning and their color (e.g., red written in green ink) to assess the cognitive inhibition manifested by the delay in the processing time (MacLeod 2005). The emotional Stroop task is a modified version of the original Stroop task in which some of the stimuli contain emotional meaning (i.e., pain-related information) to assess attentional bias toward these stimuli (Williams et al. 1996). The Stroop and Posner tasks involve different attentional processes (Cisler et al. 2009), and the Stroop task calls many processes (e.g., automatic, strategic, facilitated attention, disengagement) into play when used (Snider et al. 2000; Wright 2017). To assess attentional bias among the Arabic-speaking population, we used both an emotional Stroop task and a modified Posner cueing task to capture the different attentional processes they can measure. We excluded the dot-probe task due to time limitations and its questionable reliability (Chapman et al. 2019).

Language comprehension is another challenge in experiments including different cultures. Although images have been used to overcome cross-cultural barriers, using words has been found to be more effective than pictorial stimuli (Crombez et al. 2013; Carleton et al. 2020). Thus, it is essential to develop pain word stimuli in Arabic for use in experiments. While a considerable number of previous reaction-time-based studies present pain-related cue words for 500 ms, some literature suggests that the Arabic language processing might take a longer time to process (Bentin and Ibrahim 1996; Farghaly and Shaalan 2009). Thus, a longer cue exposure was used in the current study to ensure sufficient processing time for the Arabic words.

The current study also explored resilience levels and perceived stress among individuals with CP. Resilience is the ability to adapt positively or to preserve or reach mental health again despite facing calamity (Herrman et al. 2011). Resilience involves coping with undesired chronic circumstances (i.e., CP tolerance) (Sturgeon 2016). Despite the role that resilience plays in alleviating CP (Yeung et al. 2012), the potential moderating role of resilience has not previously been explored in relation to experimental attention tasks. Further, to explore whether participating in the tasks might produce negative consequences (i.e., the internal feeling of distress) or positive, motivating consequences (i.e., eustress) (Brulé and Morgan 2018), this study investigated perceived stress following the experimental tasks. This was particularly relevant because tasks similar to the research tasks can be used for attentional bias modification, that has the potential to improve the management of CP, by training people to reorient their attention away from pain cues (Sharpe et al. 2012; Carleton et al. 2020).

This current study tested the attention-CP link using two tasks (i.e., Posner cueing task and emotional Stroop task) that involve assessing different processes, in a new population recruited across two countries. As introduced earlier, the emotional Stroop task measures selective attention (i.e., participants must ignore the threat and attend to the color), while the Posner task measures the dynamics of attention. Regarding the current study aims, the first aim was to examine whether individuals with CP show different patterns of attentional processing of pain-related information compared to individuals without CP using different experimental tasks (i.e., Posner cueing task and modified emotional Stroop task). The second aim was to assess whether psychological resilience moderates the attentional processing of pain-related information in individuals with CP. Finally, we explored whether perceived stress levels differed between groups following participation in the attentional tasks that involve pain-related information, while controlling for baseline measurements. Additionally, this study aimed to develop a list of pain-related and neutral words stimuli that could be used in future research with the Arabic population.

## Methods

### Participants

One hundred and sixteen participants were recruited through online advertisements and posters hung at pain clinics, physiotherapy centers, hospitals, and community centers. Interested individuals contacted the researcher through a project account or email and were sent the participant information sheet. Potential participants who identified themselves as having CP were contacted and checked for inclusion criteria. The inclusion criteria for the CP group were a self-reported primary diagnosis of any CP subtype, 18 years or older, identifying themselves as having Arabic as their native language, can read and write in Arabic, being able to use a laptop, having normal or corrected to normal vision, and living in either Jordan or the United Kingdom (UK). The exclusion criteria were participants who had pain for less than three months or were medically unstable. The healthy control group were participants who did not report pain or only had mild pain on the day of the experiment. Participants gave informed consent at the start of the supervised experimental session (online or in person) and were compensated for their time according to the guidelines of UoM for reimbursement. The research project was approved by the university of Manchester research ethics committee (UREC) (ethics approval number; 2022-11074-21987) and the Jordanian ministry of health (MOH) (ethics approval number; Moh/REC/2021/233). Recruitment stopped once the target sample was reached.

The sample size was calculated according to the critical effect (i.e., the interaction of 3 predictors which are group type, cue condition, and word type) of the Posner task, which has the highest number of factors. Multiple regression model general sample equation was used by comparing sample size in other high-quality studies from a systematic review and meta-analysis conducted by the research team (i.e., 50 + 8 k) (Abudoush et al. 2021). To avoid any disruption affecting the study during the COVID-19 pandemic, a twenty-five percent dropout ratio was added to the sample totalling a hundred participants (i.e., 50 for each experimental group arm; Faul et al. 2007). The participant groups consisted of fifty-eight individuals with CP and fifty-eight healthy controls matched for age, gender and country of residence (Appendix 7 in Supplementary material). The CP and the healthy groups did not differ significantly in age; and were matched for gender and country of residence, with slightly higher recruitment from Jordan (*N* = 33) compared to the UK (*N* = 25) per group. There were no significant differences between groups in education and income level, while significant differences were found between groups on marital status, with the CP group having more married participants (Appendix 7 in Supplementary material).

### Questionnaires

An online version of self-reporting tools was embedded in the experimental procedure. Participants in both groups completed Arabic versions of the following scales online, presented according to their chronological appearance in the session.

#### Pre-experiment questionnaires

##### The Perceived Stress Scale (PSS-14) pre-test

The PSS-14 comprises 14 items that measure perceived stress, with seven positive items (i.e., 4,5,6,7,9,10, and 13) and seven negative items (which are reverse scored), with a total score is out of 56. No cut-off point is used for this scale (Cohen et al. 1994). The scale uses the 5-point Likert scale ranging from “Never” to “Always”. The Arabic-validated version has Cronbach's alpha coefficients of 0.80 (Almadi et al. 2012). This scale was also used post-experiment.

##### The Short-form McGill Pain Questionnaire (SF-MPQ)

SF-MPQ is a common tool that measures qualitative and quantitative pain characteristics. This tool consists of a 15-item checklist, divided into 11 items that assess sensory pain (throbbing, shooting, stabbing, sharp, cramping, gnawing, hot-burning, aching, heavy, tender, and splitting), and four items assess the affective dimension of the pain (tiring-exhausting, sickening, fearful, and punishing-cruel). The 15 items are rated on a 4-point pain Likert scale, where (zero) means “no pain” and (3) means “severe pain” (Melzack 1987; Terkawi et al. 2017). This questionnaire was completed by the CP group only using the Arabic-translated version with Cronbach's alpha coefficients of 0.85 (Terkawi et al. 2017).

#### Post-experiment questionnaires

In addition to the PSS-14 (post-test), the following scales were applied:

##### Connor–Davidson Resilience Scale-10 (CD-RISC-10)

The CD-RISC (Connor and Davidson 2003) has an excellent psychometric rating that measures psychological resilience with Cronbach's alpha coefficients reaching 0.85 (Campbell‐Sills and Stein 2007; Windle et al. 2011). It has ten items with a Likert scale. A higher score on this scale indicates a higher resilience rate, with forty points as the maximum possible points. An Arabic-validated version of this scale is used in this research (Toma et al. 2017).

##### Patient health questionnaire (PHQ-9)

The PHQ-9 is composed of 9 items that are used to assess depression severity (Kroenke et al. 2001). The scale has very good validity and reliability (Costantini et al. 2021). The Arabic version of this study was translated and tested for validity and reliability by Sawaya and her colleagues (2016), with Cronbach's alpha coefficients reaching 0.86 (AlHadi et al. 2017). The score of this scale range between “0” which means “never”, and “3”, which means “almost every day”. The maximum score is 27, with 5, 10, 15, and 20 cut-off points reflecting mild, moderate, moderately severe, and severe depression symptoms.

##### Generalized anxiety disorder (GAD-7)

The GAD-7 scale contains seven items that assess the severity of the anxiety symptoms, which has high validity and reliability rates (Spitzer et al. 2006). The Arabic version of this scale was translated and validated by Sawaya et al. (2016), with Cronbach's alpha coefficients reaching 0.76 (AlHadi et al. 2017). The score of this scale ranges between “0”, which means “never”, and “3”, which means “almost every day”. The maximum score is 21, with 5, 10, and 15 representing the cut-off points for mild, moderate, and severe anxiety symptoms.

### Stimuli

The list of pain-related words was obtained from the study by Harrison (1988) to ensure the validity of these translated pain-related words (i.e., sensory and affect pain-related words). We used 10 sensory pain-related words and 10 affect pain-related words for this study (Appendix 6). The neutral words were adapted from the study by Fashler and Katz (2014). The neutral words were translated using the Oxford Arabic dictionary (Arts 2014). Two independent researchers who speak Arabic and English reviewed the translations of the 10 neutral words and agreed.

### Piloting experimental tasks

Before data collection, the prepared experiment was piloted with a non-Arabic-speaking sample (*N* = 10) to ensure that it captured the basic differences between cued and uncued trials. The cueing effect direction (uncued minus cued) was negative showing an IOR effect. Further, an Arabic-speaking person with CP was invited to give comments, opinions, and feedback about the experimental session according to the patient and public involvement (PPI) principles. Their feedback and comments helped improve the experience of the experiment (e.g., the instructions, the number of breaks and their allocation).

### Modified Posner cueing task

**Fig. 1:**
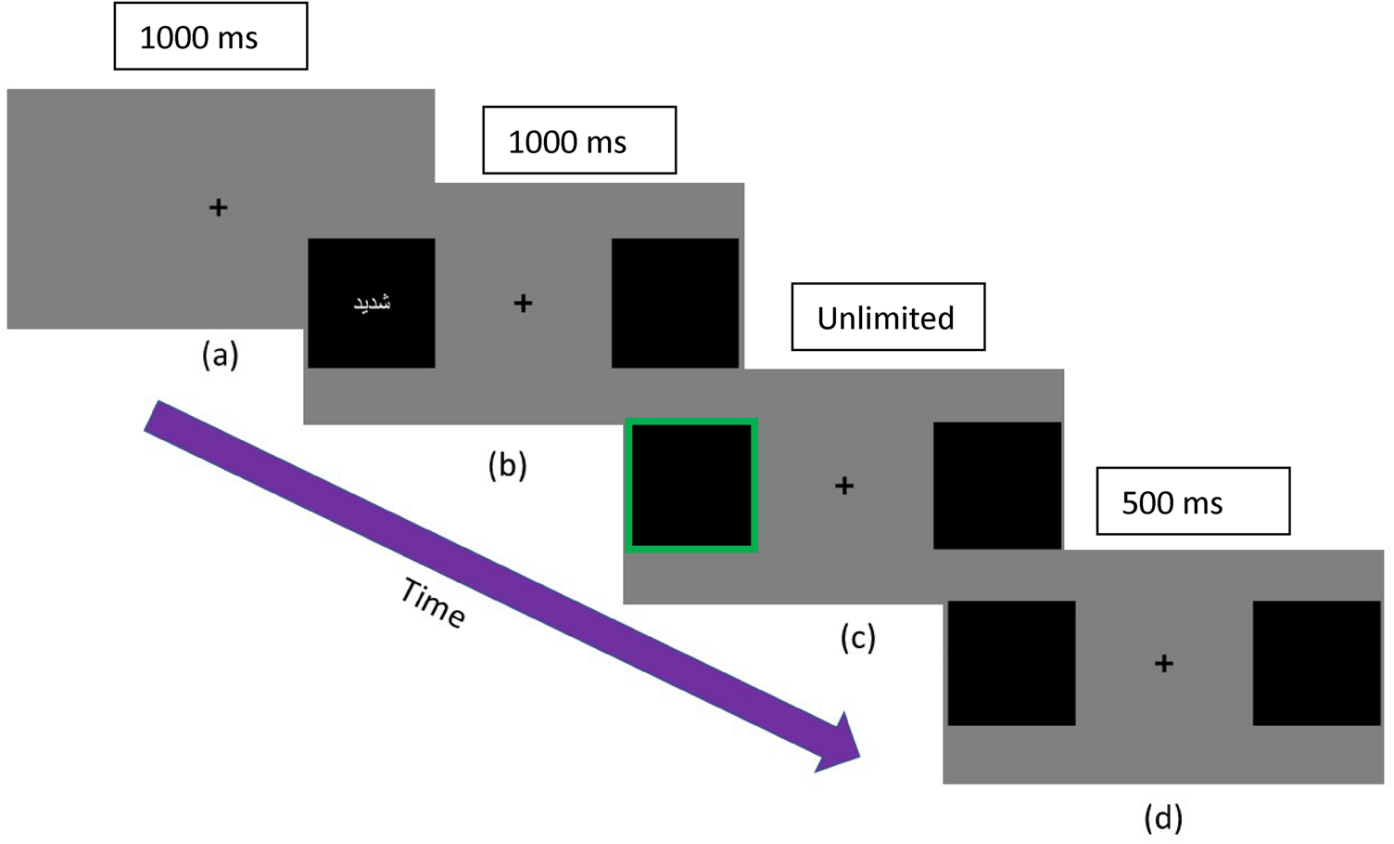
The Arabic version of the modified Posner task; **a** central fixation (1000 ms), **b** cue (1000 ms), **c** target (unlimited time), and **d** inter-trial interval (500 ms). Participants were told to respond as quickly as possible according to degree of the color of the target box frame (i.e., pressing “up” key for light green and “down” key for dark green)

Participants received detailed instructions before completing the practice trials (*N* = 10) using a neutral word to ensure they were familiar with the task. In the Posner task, a black cross fixation “ + ” first appeared against a gray background in the middle of the screen for 1000 ms. Two black squares (10 × 10 cm) presented horizontally, with the cue words—presented in white color—appearing in the middle of one of them for 1000 ms. The target—a green-colored frame on the outer edge of either the right or left black square—appeared in the same (cued) or opposite (uncued) place as the cue word with equal probability (Fig. 1). Participants were told that the cues were not predictive of the target location. This resulted in six possible conditions (i.e., cue condition (cued, uncued) × word type (sensory, affect, neutral). Twenty trials were presented per experimental condition in a randomized order. Thus, each word appeared four times (i.e., appeared in two conditions). For each of these conditions, there were four possibilities Target location (left, right) × Target color (dark, light) which occurred the same number of times in each experimental condition.

Participants were asked to determine as quickly and accurately as possible whether the color of the green frame was light green (up arrow) or dark green (down arrow) using buttons on their laptop keyboard, while concentrating on the middle of the screen and not moving their eyes or body toward the target stimulus (overt attention). The target remained on the screen until a response was given. An interval of a gray background, two black boxes and a black cross was presented for 500 ms before the next trial began. An optional break was offered before starting the task, after finishing half of the trials in each task (i.e., after 60 trials), between tasks, and after finishing the second task.

### Modified emotional Stroop task

**Fig. 2:**
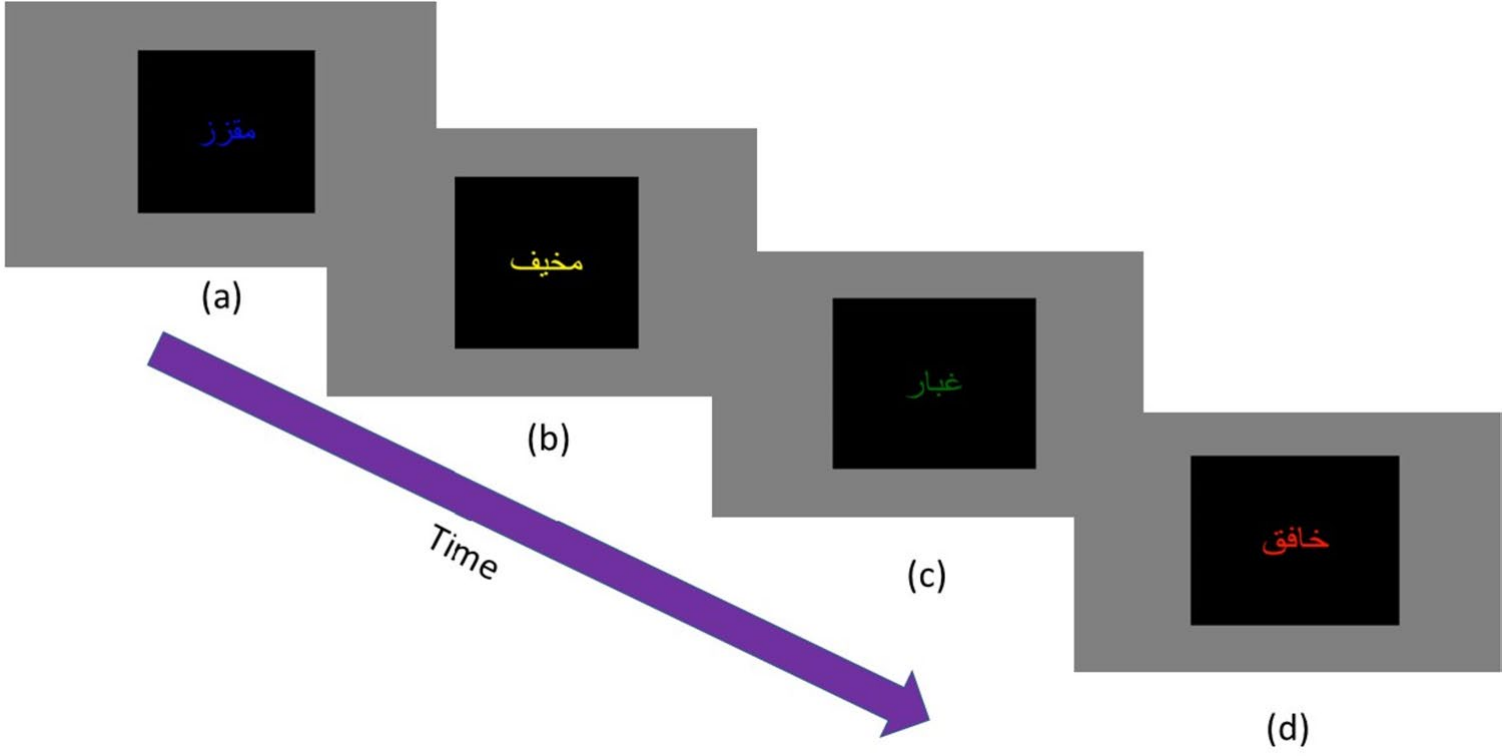
The Arabic version of the modified emotional Stroop task, **a** Sickening, **b** Fearful, **c** Dust, and **d** Throbbing. This shows an example of the temporal progression between randomized trials for word types and color presented. Participants were instructed to identify and respond to the color of the word as quickly as possible

Following instructions, participants completed practice trials (*n* = 20) using a neutral word to ensure they were oriented and familiar with the task. A modified design of the emotional Stroop task by Ben-Haim et al. (2016) was introduced to the participants. Each trial started with a single black square (10 × 10 cm) in the middle of the screen, with a colored cue word (i.e., red, green, yellow, or blue) in the middle. This square appeared on a gray background. Participants were instructed to respond to the color of the cue word by pressing either the up arrow for red color, the down arrow for green color, the right arrow for yellow color, or the left arrow button for blue color. The square and word stayed on the screen until the participant responded. Then, the subsequent trial started immediately. In total, there were 120 trials in which each of the thirty words (i.e., sensory, affect, neutral) was presented in all four possible colors. The order of presentation of the trials was randomized for both word types and colors (Fig. 2).

### Procedure

The study was pre-registered on the open science framework (OSF) (Abudoush et al. 2021) at the beginning of the data collection and before any data had been checked. The research was conducted using a hybrid approach; that is, both remote supervised sessions and face-to-face research sessions were used depending on the timing and participant. Both approaches used the same experimental research and the same software to run the experiment on the Pavlovia platform (Peirce 2007). An online remote data collection session was offered to begin with, especially at the beginning of the recruitment process due to COVID-19 restrictions. Then, in-person sessions were added to the recruitment process while offering a remote data collection session first. All participants were encouraged to use a screen with a standard size of 15.6 inches and a 1920 × 1080, 80 Hz display where possible.

For the face-to-face sessions, the COVID-19 regulations were followed carefully to avoid contamination or infection, including using personal protective equipment. Participants were encouraged to bring their laptops, when possible, to decrease the possibility of infection. Otherwise, an Acer laptop was used with a 15.6-inch screen, with a Windows 10 operation system, 1920 × 1080 pixels, 80 Hz. After ensuring that all regulations were met and setting the device for the experiment, the participant started the experiment with the examiner behind the participant. A pre-prepared mirror helped the examiner monitor and ensured that the participant's eyes focused on the middle of the screen.

For the remote sessions, one day before the experiment, the researcher sent an invitation link to the participant and ensured the appropriateness of the place where the research session will be conducted. Participants were instructed to use a laptop with a Windows operating system (version 7 or later), with the Zoom application installed on it (Zoom Video Communications Inc. 2022), and to choose a suitable place which has only the participant at the time of research, limit noise as possible, and reduce light and/or sit away from the window to avoid glare on the laptop screen. At the beginning of the research session, the researcher welcomed, explained and checked that the setting was appropriate (i.e., minimize light, avoid glare on the screen, quiet and private room, and ensure that the device is around 60 cm distance from the participant) for the research session to start. Then, the researcher turned off his camera and muted the mic to avoid distractions while the participant was completing the experimental session. The participant camera stayed on, so the researcher supervised and ensured that the participant was engaging in the experiment and that participant`s eyes were focusing on the middle of the screen during the experimental tasks. Using the Zoom chat box, the researcher then put the experiment’s link and instructed the participant to use it to start the experiment.

For both types of sessions, the instructions embedded in the experiment guided the participant using PsychoPy software pushed to the Pavlovia platform (Peirce 2007; Peirce et al. 2019). Regular breaks were offered to participants during the research session, and the researcher checked that participants were comfortable or had anything they wanted to mention or ask about. The chronological order of the experiment started with an automated consent form, then questions about demographics (i.e., age, gender, education, social status, marital status, and country of residence). The CP group also answered the MPQ-SF at this point. Then, this was followed by answering the PSS scale and then the two experimental tasks (i.e., Modified Posner cueing task and Modified emotional Stroop task). The order of the presentation of the tasks was counterbalanced, and the trials in each task were randomized. After the experiment, the PSS-14 was presented again, assessing the pain severity using a numeric pain scale (i.e., as a sub-scale of the MPQ-SF scale). Next, the CD-RISC-10 scale was used to assess the resilience level, followed by the PHQ-9 to assess the depression symptoms level and the GAD-7 to assess the anxiety symptoms level. At the end of the experimental tasks and questionnaires, the last task was to evaluate on a Likert scale out of five (i.e., from “1”, which means not appropriate to “5” were very appropriate) whether each word used in the experimental tasks was appropriate and understood from the participant’s point of view to be used in similar future experimental studies related to pain in general.

After finishing the experiment, the participants from the CP group who provided additional consent were interviewed about the experiment and CP-attention-related experiences. The qualitative results were analyzed and published in a separate paper (Abudoush et al. 2023a, b). The total time of the research session lasted around 60–90 min for the CP group and around 60–70 min for the healthy control groups.

### Analytical plan and data handling

The data were cleaned and tidied using the R software version (Team, 2022) following the pre-registered analysis plan on the OSF and deviations from this are noted in the results section. No participant data was lost or missed during the data collection session. All analyses were done using RStudio software (RStudio Team 2020). The number of trials and participants removed at each step is presented in Appendix 1 (in Supplementary material). One participant from the healthy group had more than 30% wrong answers on both experimental tasks and was consequently excluded from further analysis as planned in the pre-registration. Outliers were removed according to the pre-planned range (i.e., > 3000 ms or < 250 ms) since responses outside this window would indicate anticipation or a lapse in concentration. Trimming of data was done using the interquartile data normalizing equation (i.e., Q1 − 1.5*IQR, Q3 + 1.5*IQR) for each condition separately; then, participants’ data with more than 30% data loss on all conditions in a particular task or 50% from a particular condition were excluded. This second rule was developed after the pre-registration, when looking at overall error rates, but prior to any statistical analysis. The data were checked for normality using plotting (QQ normal and density plots) and the Shapiro–Wilk test and log-transformed when normality was violated. Because of the nature of the data collected (i.e., different levels of variables), an additional analysis of a multiple linear random mixed-effect regression model was used for some analyses.

## Results

For comorbid symptoms, anxiety symptoms were mild in the CP group, with a significant difference between groups. Depression symptoms were also mild in the CP group, with a significant difference between groups. On the SF-McGill pain questionnaire, the average pain intensity was severe in the CP group in the pre- and post-experiment measurement. The CP group described their pain mainly as being “distressing” to “horrible”. The duration of the pain ranged between 4 months and 24 years (see Appendix 8 in Supplementary material). Ratios of CP subtypes are summarized in Appendix 2 (in Supplementary material). The overall mean response on the severity of subscales of the pain words (i.e., subscales words in the McGill questionnaire) for the CP group was moderate on both sensory (*M* = 1.50, SD = 1.18) and affect words (*M* = 1.86, SD = 1.16). Mean responses for each word are summarized in Appendix 3 (in Supplementary material). The results from the analysis addressing each study aim are described in the next sections.

### Do individuals with and without CP show different patterns of attentional processing of pain-related information using different experimental tasks?

To test whether there were between-groups differences on the Posner task (aim one), a three-way analysis of variance (ANOVA) test [group type, cue condition, word type] on reaction times revealed a significant main effect of group *F*(1, 671) = 19.97, *p* < 0.001 with the CP group being slower overall. However, there was no significant main effect of cue condition or interaction between word type and cue condition (Appendix 4, Table 1 in Supplementary material).

ANOVA analysis can reveal basic differences between different stimuli and word types, but the random linear mixed effect model can account for more variance related to the word types. Thus, it was informative to add this to the planned analysis (Appendix 4, Table 2 in Supplementary material). Analysis revealed significant overall differences between groups *t*(140) = 2.43, *p* = 0.0166, and significant differences between groups on sensory stimuli compared to neutral stimuli *t*(580) = 2.61, *p* = 0.009. The overall cueing effect was significant *t*(583) = 3.22, *p* = 0.001. The cueing effect direction (uncued minus cued) was negative showing slower responses to cued than uncued stimuli (IOR). No significant differences were found between groups on the affect stimuli compared to neutral stimuli *t*(581) = 0.52, *p* = 0.606. Regarding the covariates, there were overall significant effects of age *t*(101) = 5.99, *p* < 0.001 (i.e., longer reaction time for older participants) and gender *t*(102) = 3.00, *p* = 0.003, (i.e., longer reaction time for males) but not for education, income level, marital status, or country of residence (Appendix 4 in Supplementary material). The overall model R squared (0.88). The best-fit model was used according to the Akaike information criterion (AIC) value of − 1063.52, and the Bayesian information criterion (BIC) value of − 950.36 (Burnham and Anderson 2004).

**Fig. 3:**
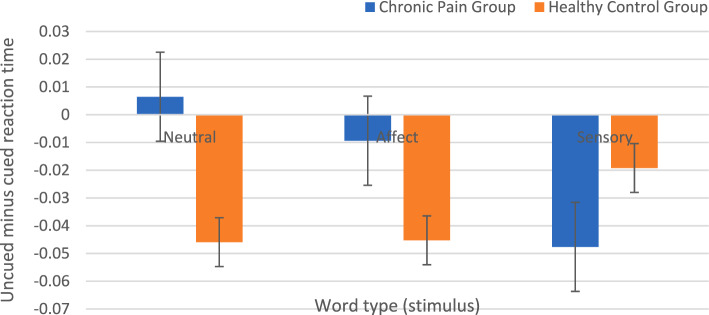
Mean (standard error of the mean) of the uncued minus cued reaction time difference for the CP and healthy control groups in the sensory, affect, and neutral words conditions. The CP group exhibited IOR only for sensory words, while the control group exhibited IOR (i.e., faster uncued than cued RTs) for all word types

Between-group t-test and within-group paired t-test mean comparisons for the cueing effect were conducted to assess the specific groups' differences (i.e., uncued minus cued reaction time) as illustrated in Fig. 3, and summarized in Appendix 9 (in Supplementary material). The CP group took more overall time to react to a neutral stimulus compared to sensory stimulus *t*(112) = 2.44, se = 0.022, 95% CI [− 0.098, − 0.01], *p* = 0.016 with mean of difference *m* = − 0.054, reflecting a longer response to neutral stimuli (i.e., mean sensory—mean neutral = − 54 ms). There was a non-significant difference between CP and healthy groups and a trend for the neutral and affect words (Appendix 9 in Supplementary material). The control group exhibited IOR (i.e., faster uncued than cued RTs) for all word types with no significant differences, whereas the CP group showed IOR only toward sensory words; the cueing effect differed significantly between sensory and neutral words for the CP group. Bonferroni correction (i.e., *p* ≤ 0.0167) was used to assess for potential type I error and confirmed the significant differences between the sensory and neutral stimuli in the CP group (Appendix 9 in Supplementary material).

To assess between-groups differences on the Stroop task (aim two), an ANOVA [Group × Word Type] on reaction time revealed significant differences between groups *F*(1, 312) = 8.00, *p* = 0.005, with CP group displaying a slower overall reaction time compared to healthy controls (Appendix 4, Table 3 in Supplementary material). There was no significant main effect of word type *F*(2, 312) = 0.21, *p* = 0.812, or group × word interaction *F*(2, 312) = 0.05, *p* = 0.954 confirming the null hypothesis.

Pearson correlations were used to explore correlations between the two tasks (cueing effect for Posner and reaction time for Stroop) for each word type (aim three). This revealed a non-significant weak negative correlation between affect words *r*(51) = − 0.16, *p* = 0.258. Non-significant correlations were found for sensory words *r*(50) = 0.07, *p* = 0.617, and neutral words *r*(51) = − 0.08, *p* = 0.550 (Appendix 5 in Supplementary material).

### Does psychological resilience modulate the attentional processing of pain-related information in individuals with CP?

An independent samples t-test revealed a significantly lower resilience level in the CP than the healthy control group *t*(111) = − 2.56, 95% CI [− 4.95, − 0.63], *p* = 0.012 (Appendix 10 in Supplementary material).

To assess the modulatory effect of resilience on the Posner and Stroop tasks (aim two), a multiple linear random mixed-effect regression model was used. An interaction factor term (i.e., group type*word condition*resilience score) was made for assessing the moderation effect of psychological resilience level on participants’ performance on the Posner task (i.e., using cueing differences between uncued minus cued condition as the dependent variable) and revealed a significant between-group effect *t*(217) =  −2.17, se = 0.003, *p* = 0.031 for sensory words compared to neutral words, while the effect was non-significant *t*(214) = − 1.77, se = 0.003, *p* = 0.079 for the affect words. Overall, the CP group showed lower cueing effects and lower resilience scores compared to the healthy group, mirroring the previous analysis. Healthy controls showed an overall positive association between the level of resilience and cueing effect for sensory and affect words, which was less obvious in the CP group (Appendix 11 in Supplementary material). The proportion of the variation in the dependent variable explained by the independent variable (i.e., *R* squared) reached 0.49, with AIC = − 471.86 and BIC = − 418.34. For the emotional Stroop task, the interaction with resilience was not significant for either sensory *t* (204) = 0.503, se = 0.003, *p* = 0.616 or affect words *t*(204) = − 1.106, se = 0.003, *p* = 0.915.

### Effects of participation in the attentional tasks on perceived stress levels

To assess between-groups differences in perceived stress post-experiment, an ANCOVA was used controlling for pre-experiment baseline measurement and other covariates. Differences between experimental groups were significant *F*(1,100) = 7.40, *p* = 0.008, with the CP group having higher perceived stress scores than healthy controls. However, the interaction between group type*pre-test was non-significant *F*(1,100) = 0.27, *p* = 0.604, indicating that post-test differences could be explained by pre-experiment differences. The effects of all other covariates controlled for (i.e., age, gender, education, country, income level, and marital status) were non-significant.

### Evaluation of word stimuli

Both groups evaluated the words used in the study at the end of the experiment for the CP group (*M* = 4.28, SD = 1.60) with mean appropriateness of 85.6% (i.e., 4.28 out of 5), and the healthy control group (*M* = 4.26, SD = 1.66) mean appropriateness of 85.2% (i.e., 4.26 out of 5). The descriptive statistics for the words are listed in Appendix 6 (in Supplementary material).

## Discussion

This study examined attentional biases in relation to pain in the Arabic population with and without CP. The association between resilience and attentional performance, and the perceived stress among this population were also explored. Regarding the first aim of the study, the analysis of results based on the overall reaction times from the Posner task showed that the CP group did not respond more quickly to words related to sensory pain-related stimuli than the control group. However, they responded more quickly following sensory cues than following neutral cues, which has not been reported in the previous literature (Crombez et al. 2013; Abudoush et al. 2023a, b). This difference might have resulted from the tendency of CP participants to disengage early from sensory information as will be discussed below. One previous study that used exogenous cueing task showed that responses of patients with irritable bowel syndrome (IBS) did not differ between neutral and pain-related stimuli (Chapman and Martin 2011).

Because the cue presentation time was relatively long, a negative cueing effect was observed (i.e., IOR effect; Li et al. 2017). While healthy controls showed IOR across the different cue stimuli, the CP group exhibited IOR only for the pain-sensory stimuli. IOR is proposed to result from participants disengaging from the cued location and becoming faster to respond to stimuli presented at the uncued location. IOR usually occurs when an exogenous sensory stimulus is presented at a peripheral visual-spatial location when the eyes are still and is attributed to a delay in attentional reorientation to the previously cued location (Klein 2000). Thus, the CP group showed delayed disengagement for the neutral and affect stimuli and faster disengagement (i.e., IOR emerged) for the sensory stimuli. This suggests that the nature of the cue affected the timing of the disengagement process, as seen for participants with non-clinical somatoform dissociation in a study using tactile stimuli (Brown et al. 2010).

This study applied a slightly later time point than most other studies and found an IOR effect, therefore the findings in this study differ from previous hypotheses related to the vigilance–avoidance pattern stemming from experiments in the anxiety field (Mogg and Bradley 1998; Mogg et al. 2004; Todd et al. 2015). Also, using 50% valid cues we were more likely to observe IOR. Previous studies that explored long presentation time points using non-predictive cues (i.e., 50%) did not detect an IOR effect, but this is likely to be due to using a dot-probe rather than a Posner task (Liossi et al. 2010; Schoth and Liossi 2013; Garland and Howard 2013; Fashler and Katz 2014; Mazidi et al. 2019). Because there were no similar previous studies in the Arabic population, a comparatively longer presentation time (i.e., 1000 ms) was chosen due to language processing differences (Bentin and Ibrahim 1996; Farghaly and Shaalan 2009). However, the fact that an IOR effect was produced in most conditions in the control group indicates that the Arabic language might not need longer processing time as previously thought. Thus, future research should aim to replicate the study with more than one presentation time (i.e., > 500 ms and < 500 ms) to explore both earlier and later components of attentional orienting. Further, replication is important as this effect was observed in the exploratory analysis.

Regarding between-groups differences in the emotional Stroop task, the CP group exhibited a slower overall reaction time compared to healthy controls, but these differences were non-significant. This non-significant trend may relate to general cognitive or dexterity issues or it may be that the inclusion of pain-related stimuli in the experiment slowed participants down. The fact that there were no differences in how the groups performed across the word conditions in the Stroop may be due to the different attentional processes involved compared to the Posner task. Indeed, there was no correlation between the two tasks. This might be expected given that these experimental tasks measure different attentional processes (Cisler et al. 2009). It is interesting to note that although the Stroop task involves multiple attentional processes and therefore it may be more likely to observe group differences for this task, it appears from the current results that important group differences may be present in the dynamics of spatial attention.

For the CP group, only the sensory pain words produced disengagement (i.e., IOR) and a similar pattern to that seen mainly across neutral and affect cue words for the control group. Thus disengagement was delayed for the neutral and affective words in the CP group. This finding suggests a potential change in information processing among the CP group with relatively long cue presentations in this population. The CP group may have attended more (i.e., hypervigilance) to the neutral and affect words, searching for the presence of threat, which caused delayed disengagement. This appears to have been overridden for the sensory information because of the CP, which led to faster disengagement. These findings suggest that the CP group might show avoidance of sensory stimuli, which aligns with some previous models that explained the attentional processes in the context of CP (Abudoush et al. 2023a, b). However, it is unclear why affective pain-related information did not produce a similar early engagement in the CP group, given that affective experiences trigger the same areas in the neural system. The somatic experiences generated by the sensory pain words might explain this difference. Because of their chronic situation, the CP group showed avoidance of sensory stimuli because of their experiences (Chapman and Martin 2011; Satpute et al. 2015), resulting in the control group showing greater engagement with sensory stimuli as it is not the normal situation for them. The evidence around the affective pain-related information is a related to idiosyncratic cognitive reasoning and still at a preliminary evidence level, which could partially justify the more salient effect of the sensory information (Patterson et al. 2012; Abudoush et al. 2023a, b). Thus, further experimental research is needed to explore this phenomenon in different paradigms and different stimuli.

The second aim of the study focused on the role of resilience. As expected, the CP group had significantly lower resilience than healthy controls, consistent with previous findings that CP is associated with low resilience and higher distress levels (Goubert and Trompetter 2017). Interestingly, resilience was found to modulate performance in the Posner task. Healthy controls showed an overall positive direction of effect for the level of resilience and cueing effect (i.e., sensory and affect words). This means higher resilience was associated with a lower magnitude of (more positive) IOR and potentially later disengagement. However, the direction of the effect was not clear for the CP group regarding the affect pain-related cues but showed that extreme resilience levels (i.e., very low or high) were associated with higher magnitude of (more negative) IOR for sensory pain-related cues (Appendix 11 in Supplementary material). The results suggest that individuals with CP may avoid (or disengage early from) distressing information instead of facing them because of either high avoidance pattern (i.e., low resilience) or flexible management (i.e., high resilience) when exposed to threat (Crane and Searle 2016). Although the distress avoidance pattern is usually found in people with lower resilience, it is also salient in individuals with CP (Ramírez-Maestre et al. 2014). Future studies should further explore the spatial cueing–resilience association over several time points and in different populations. Furthermore, integrated interventions that include using attentional bias modification that trains attention away from pain cues (Sharp et al. 2012; Carleton et al. 2020) could be promising in re-orienting attention when exposed to distress-related situations.

In relation to stress, significant differences were found between groups at baseline (i.e., higher perceived stress in the CP group compared to healthy controls). Perceived stress levels did not differ significantly between groups after the experiment while controlling for baseline. These results indicate that exposure to pain-related information within a research study is not unduly distressing in the Arabic population, setting the scene for potential future studies with this population. This suggests it is reasonable and ethical to use these approaches, and it suggests that attentional bias modification tasks would also be reasonable to use within interventions. This was also consistent with the findings from the qualitative data (Abudoush et al. 2023a, b).

A strength of this study was the involvement of an individual with a CP in the research development who provided feedback that enhanced the presentation of the experiment and improved the overall experience of the participants during the experimental session. Public involvement became a more common practice in recent research studies in the cognitive neuroscience field (Sullivan and Poliakoff 2023). Additionally, participants from both groups evaluated the words used in the experiment revealing that they found the words to be highly appropriate. This Arabic word list can be used for future research on this population.

The design of this hybrid experimental study provided a flexible approach that overcame health restrictions, and distance barriers enabling recruitment from two countries. Controlled experiments can be accessible, and feasible with this methodological design. The advantages, disadvantages, designing, and implementation of online behavioral experiments, such as easy access and cost-effectiveness versus difficulties related to controlling the experimental environment, have been discussed within the broader experimental psychology literature (Sauter et al. 2020; Zaadnoordijk and Cusack 2022). Future research should consider the controlled hybrid and remote application of the experiments done in this study as an effective, cost-efficient, and more accessible option to CP individuals, especially those living in distant, less fortunate areas. Such methodologies ensure that experiments are controlled, yet can be accessible by the target population. Also, it is a feasible method to deliver interventions.

Despite the strengths of this study, limitations should also be considered. First, it was only possible to assess some attentional processes due to the late time point chosen. Comparing different time points in future studies would allow for a deeper understanding of attention mechanisms in this population. Second, the nature of the hybrid study meant it was not possible to use advanced technological tools (e.g., eye-tracking). Future studies need to take these tools into account when possible. Lastly, this study included words only as a stimulus due to the nature of the tasks involved. It is recommended that future studies investigate the effect of using other stimuli (e.g., images, video, tactile), which align with the recommendations of a recent systematic review and meta-analysis (Abudoush et al. 2023a, b).

In addition to the recommendations mentioned above, it is important to assess the attentional biases of Arabic individuals with CP—and other CP populations—through other types of stimuli, such as pictorial stimuli. Using eye tracking as well as reaction times would also enable a more continuous measure of attentional processing. Because attentional biases appear to be involved in the difficulties that affect the functionality and quality of life of individuals with CP (Todd et al. 2015), developing easy access and reliable assessment procedures is crucial. This would enhance establishing an agreed cross-cultural format of experiments and settings. Finally, the pre-registration of this study ensured the high quality of the methods used. It also recruited open-access technologies that made the experiment reproducible. Many previous studies needed a similar approach, which affected data availability and reproducibility (Abudoush et al. 2023a, b). Thus, we recommend that future studies use such open-access reproducible designs.

## Conclusion

This first experimental study of attentional biases in the Arabic-speaking population with and without CP revealed that it is feasible to measure attentional processes in this population using a hybrid methodology, without causing undue stress to participants. This paves the way for future research in the field of SA-CP among Arabic participants, which could benefit from the findings and recommendations made. The healthy control group showed IOR across cue conditions, while the CP group was influenced by the condition, with the IOR effect only evident in sensory pain-related information condition. This suggests that the timing of disengagement of attention is affected in CP, which should be investigated further. Resilience levels in the CP and control group moderated the performance on the Posner task, suggesting that resilience might play an important role in attentional performance. Finally, the findings suggest that disengagement ought to be investigated in different CP populations.

### Supplementary Information

Below is the link to the electronic supplementary material.

Supplementary file1 (DOCX 235 KB)

## Data Availability

Supplementary materials included. Other non-identifying data can be provided on request.
