# Supplementary material for: Investigating attention toward pain-related cues in an Arabic-speaking population with and without chronic pain
Source: Exp Brain Res. 2024 Feb 29;242(4):843–55. doi: 10.1007/s00221-024-06789-9 (PMC10972906; doi:10.1007/s00221-024-06789-9)
Supplement: Supplementary file 1 — Supplementary file1 (DOCX 235 KB) [file 221_2024_6789_MOESM1_ESM.docx]

**Appendix 1: The ratio of data removed from through cleaning steps**

Table 1: Trimming data for the Posner task

| **Cleaning step** | **Chronic pain group** | **Healthy control group** |
| --- | --- | --- |
| Removing wrong answers (>30%) | NA | N=1 (1.72%) |
| Removing answers out of the pre-determined attention spectrum (<.25 or > 3.0 sec) | 2.95% | 1.52% |
| Trimming using interquartile equation | 4.62% to 6.87% | 3.56% to 4.70% |
| Removing data from tasks with 30% lost data on all conditions | NA | NA |
| Removing data from tasks with 50% lost data for each condition | 2 conditions from 2 participants | 5 conditions from 3 participants |

Table 2: Trimming data for the emotional Stroop task

| **Cleaning step** | **Chronic pain group** | **Healthy control group** |
| --- | --- | --- |
| Removing wrong answers (>30%) | NA | N=1 (1.72%) |
| Removing answers out of the pre-determined attention spectrum (<.25 or > 3.0 sec) | 7.28% | 4.34% |
| Trimming using interquartile equation | 5.72% to 5.98% | 4.90% to 5.33% |
| Removing data from tasks with 30% lost data on all conditions | N=5 | N=4 |
| Removing data from tasks with 50% lost data for each condition. | NA | NA |

**Appendix 2: Pain characteristics**

| **Type of chronic pain** | Lumbar low back pain | Cervical Neck pain | ThoracicBack pain | Headache (including migraine, tension headache, cluster headache, sinus headache) | Post-injury chronic pain (musculoskeletal, burn, | Ulcerative colitis | GERD (chronic acid reflux) | Limbs-pain Non- injury related (Osteoarthritis, overuse, ..) | **RA** | **FM** | IBS | Planter fasciitis-bone spur | |
| --- | --- | --- | --- | --- | --- | --- | --- | --- | --- | --- | --- | --- | --- |
| **Ratio Jordan** | 7  (21.21%) | 4 (12.12% | - | 5 (15.15%) | 4 (12.12%) | 1 (3.03%) | 2 (6.06%) | 5 (15.15%) | **1 (3.03%)** | - | 1 (3.03%) | 3 (9.09%) |  |
| **Ratio UK** | 6 (24%) | 2 (8%) | 1 (4%) | 5 (20%) | 6 (24%) | - | - | 3 (12%) | - | **1 (4%)** | **1 (4%)** | - |  |
| **Total ratio** | 13 (22.41% | 6 (10.34%) | 1 (1.72%) | 10 (17.24%) | 10 (17.24%) | 1 (1.72%) | 2 (3.45%) | 8 (13.79%) | 1 (1.72%) | **1 (1.72%)** | **2 (3.45%)** | 3 (5.17%) |  |

**Appendix 3: Mean responses for words on the Short Form-McGill questionnaire subscales**

| Sensory pain words | Mean | SD |
| --- | --- | --- |
| Throbbing | 1.24 | 1.26 |
| Shooting | 1.48 | 1.23 |
| Stabbing | 1.52 | 1.19 |
| Sharp | 1.93 | 1.07 |
| Cramping | 1.69 | 1.14 |
| Gnawing | 0.93 | 1.12 |
| Hot-Burning | 1.21 | 1.28 |
| Aching | 1.62 | 1.18 |
| Heavy | 1.93 | 1.12 |
| Tender | 1.72 | 1.15 |
| Splitting | 1.26 | 1.26 |
| Affect pain words |  |  |
| Tiring-Exhausting | 2.22 | 0.94 |
| Sickening | 1.62 | 1.35 |
| Fearful | 1.50 | 1.25 |
| Punishing-Cruel | 2.10 | 1.04 |

Pain words from subscales dimensions (i.e. Sensory pain words, Affect pain words) of the Mild =1, Moderate 2, Severe =3

**Appendix 4: linear random mixed effect model results**

**Hypothesis one (Assessing between-groups differences on the Posner task)**

**Table 1: ANOVA**

| **Variable** | **df** | **F value** | **P-value** |
| --- | --- | --- | --- |
| Group | 1, 671 | 19.97 | < .001 *** |
| Word | 2, 671 | 0.20 | 0.819 |
| Cue | 1, 671 | 2.55 | 0.111 |
| Group*Word*Cue | 2, 671 | 0.52 | 0.593 |

**Table 2: Linear Mixed effect model**

| **Variable** | **Estimate** | **Std. Error** | **df** | **t value** | **P-value** |
| --- | --- | --- | --- | --- | --- |
| Intercept | -3.187e-01 | 1.020e-01 | 1.036e+02 | -3.123 | 0.002** |
| Group: |  |  |  |  |  |
| - HCG | - | - | - | - | Ref. |
| - CPG | 3.015e-02 | 3.356e-02 | 1.409e+02 | 0.898 | 0.371 |
| Words: |  |  |  |  |  |
| - Neutral | - | - | - | - | Ref. |
| - Affect | -6.893e-03 | 1.430e-02 | 5.581e+02 | -0.482 | 0.630 |
| - Sensory | -2.539e-02 | 1.430e-02 | 5.581e+02 | -1.776 | 0.076. |
| Cue: |  |  |  |  |  |
| - cuedlog | - | - | - | - | Ref. |
| - uncuedlog | -4.635e-02 | 1.438e-02 | 5.583e+02 | -3.224 | 0.001** |
| Age: | 7.975e-03 | 1.330e-03 | 1.019e+02 | 5.994 | 3.12e-08*** |
| Gender: |  |  |  |  |  |
| - Female | - | - | - | - | Ref. |
| - Male | 9.351e-02 | 3.120e-02 | 1.020e+02 | 2.997 | 0.003** |
| Interaction term: |  |  |  |  |  |
| - HCG*cued*neutral | - | - | - | - | Ref. |
| - CPG*uncued*affect | -1.466e-02 | 2.839e-02 | 5.581e+02 | -0.516 | 0.606 |
| - CPG*uncued*sensory | -7.394e-02 | 2.835e-02 | 5.580e+02 | -2.608 | 0.009** |

**Hypothesis two (Assessing between-groups differences on the Stroop task)**

**Table 3: ANOVA**

| **Variable** | **Df** | **F value** | **P-value** |
| --- | --- | --- | --- |
| Group | 1, 312 | 8.00 | .005** |
| Word | 2, 312 | 0.21 | 0.812 |
| Group*Word | 2, 312 | 0.05 | 0.954 |

**Appendix 5- Matrix panel of the correlations with histograms for each task-word type**


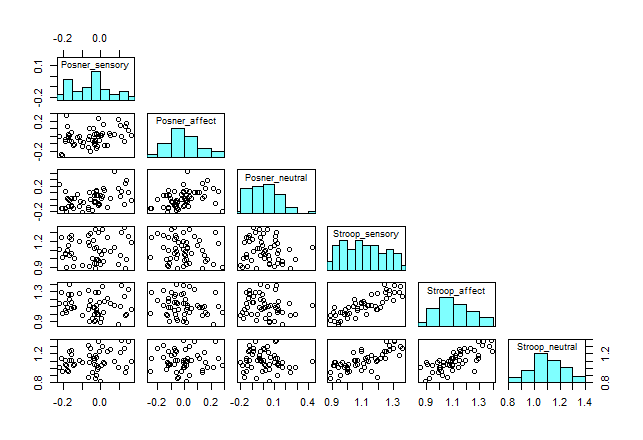


**Appendix 6: Words Evaluation**

**Table 1: Words evaluation by the chronic pain group**

| Word English | Word Arabic | Cueing type | Mean | Median | SD | LQ | UQ | IQR |
| --- | --- | --- | --- | --- | --- | --- | --- | --- |
| Pulsing | نابض | Sensory | 3.672413793 | 4 | 1.443532838 | 3 | 5 | 2 |
| Closet | خزانة | Neutral | 4.189655172 | 5 | 1.468462575 | 4 | 5 | 1 |
| Tiring | متعب | Affect | 4.827586207 | 5 | 0.464075426 | 5 | 5 | 0 |
| Throbbing | خافق | Sensory | 3.534482759 | 4 | 1.569618539 | 2 | 5 | 3 |
| Kitchen | مطبخ | Neutral | 4.327586207 | 5 | 1.394071798 | 5 | 5 | 0 |
| Exhausting | منهك | Affect | 4.534482759 | 5 | 1.029657024 | 5 | 5 | 0 |
| Beating | ضارب | Sensory | 4 | 5 | 1.337712108 | 3 | 5 | 2 |
| Table | طاولة | Neutral | 4.344827586 | 5 | 1.408641552 | 5 | 5 | 0 |
| Sickening | مقزز | Affect | 4.224137931 | 5 | 1.351322983 | 4 | 5 | 1 |
| Pounding | ساحق | Sensory | 3.913793103 | 5 | 1.405309266 | 3 | 5 | 2 |
| Blender | خلاط | Neutral | 4.172413793 | 5 | 1.488410562 | 4 | 5 | 1 |
| Suffocating | خانق | Affect | 4.431034483 | 5 | 1.077880782 | 4 | 5 | 1 |
| Drilling | لاذع | Sensory | 3.948275862 | 5 | 1.419444094 | 3 | 5 | 2 |
| Water | ماء | Neutral | 4.534482759 | 5 | 1.231395119 | 5 | 5 | 0 |
| Fearful | مخيف | Affect | 4.517241379 | 5 | 1.188266898 | 5 | 5 | 0 |
| Boring | ثاقب | Sensory | 3.810344828 | 5 | 1.616330005 | 3 | 5 | 2 |
| Vase | مزهرية | Neutral | 4.362068966 | 5 | 1.397971913 | 5 | 5 | 0 |
| Frightful | مرعب | Affect | 4.431034483 | 5 | 1.229920393 | 5 | 5 | 0 |
| Stabbing | طاعن | Sensory | 3.982758621 | 5 | 1.468874486 | 3 | 5 | 2 |
| Ladder | سلم | Neutral | 4.224137931 | 5 | 1.351322983 | 4 | 5 | 1 |
| Punishing | قاسي | Affect | 4.603448276 | 5 | 0.954024811 | 5 | 5 | 0 |
| Lancinating | يمزق | Sensory | 4.655172414 | 5 | 0.909052411 | 5 | 5 | 0 |
| Towels | مناشف | Neutral | 4.431034483 | 5 | 1.258125436 | 5 | 5 | 0 |
| Gruelling | مرهق | Affect | 4.517241379 | 5 | 1.143116382 | 5 | 5 | 0 |
| Pinching | قارص | Sensory | 3.965517241 | 5 | 1.4505355 | 3 | 5 | 2 |
| Dust | غبار | Neutral | 4.396551724 | 5 | 1.269613219 | 5 | 5 | 0 |
| Vicious | شديد | Affect | 4.603448276 | 5 | 0.990120588 | 5 | 5 | 0 |
| Pressing | ضاغط | Sensory | 4.396551724 | 5 | 1.183803788 | 4.25 | 5 | 0.75 |
| House | منزل | Neutral | 4.517241379 | 5 | 1.202940539 | 5 | 5 | 0 |
| Killing | قاتل | Affect | 4.448275862 | 5 | 1.230780865 | 5 | 5 | 0 |

**Table 2: Words evaluation by the healthy control group**

| Word English | Word Arabic | Cueing type | Mean | Median | SD | LQ | UQ | IQR |
| --- | --- | --- | --- | --- | --- | --- | --- | --- |
| Pulsing | نابض | Sensory | 3.614035 | 4 | 1.33302 | 3 | 5 | 2 |
| Closet | خزانة | Neutral | 4.263158 | 5 | 1.343321 | 4 | 5 | 1 |
| Tiring | متعب | Affect | 4.526316 | 5 | 1.053993 | 5 | 5 | 0 |
| Throbbing | خافق | Sensory | 3.736842 | 4 | 1.518062 | 3 | 5 | 2 |
| Kitchen | مطبخ | Neutral | 4.333333 | 5 | 1.327368 | 5 | 5 | 0 |
| Exhausting | منهك | Affect | 4.438596 | 5 | 1.195491 | 5 | 5 | 0 |
| Beating | ضارب | Sensory | 3.77193 | 4 | 1.427004 | 3 | 5 | 2 |
| Table | طاولة | Neutral | 4.385965 | 5 | 1.292207 | 5 | 5 | 0 |
| Sickening | مقزز | Affect | 4.017544 | 5 | 1.552547 | 3 | 5 | 2 |
| Pounding | ساحق | Sensory | 3.947368 | 5 | 1.481084 | 3 | 5 | 2 |
| Blender | خلاط | Neutral | 4.22807 | 5 | 1.464064 | 5 | 5 | 0 |
| Suffocating | خانق | Affect | 4.035088 | 5 | 1.349139 | 3 | 5 | 2 |
| Drilling | لاذع | Sensory | 3.842105 | 5 | 1.544454 | 3 | 5 | 2 |
| Water | ماء | Neutral | 4.649123 | 5 | 0.990873 | 5 | 5 | 0 |
| Fearful | مخيف | Affect | 4.631579 | 5 | 0.918687 | 5 | 5 | 0 |
| Boring | ثاقب | Sensory | 4.333333 | 5 | 1.074598 | 4 | 5 | 1 |
| Vase | مزهرية | Neutral | 4.298246 | 5 | 1.36231 | 5 | 5 | 0 |
| Frightful | مرعب | Affect | 4.45614 | 5 | 1.001252 | 4 | 5 | 1 |
| Stabbing | طاعن | Sensory | 3.982456 | 5 | 1.407775 | 3 | 5 | 2 |
| Ladder | سلم | Neutral | 3.877193 | 5 | 1.48889 | 3 | 5 | 2 |
| Punishing | قاسي | Affect | 4.473684 | 5 | 1.019546 | 4 | 5 | 1 |
| Lancinating | يمزق | Sensory | 4.140351 | 5 | 1.342154 | 3 | 5 | 2 |
| Towels | مناشف | Neutral | 4.105263 | 5 | 1.577568 | 3 | 5 | 2 |
| Gruelling | مرهق | Affect | 4.561404 | 5 | 1.000313 | 5 | 5 | 0 |
| Pinching | قارص | Sensory | 4.298246 | 5 | 1.179663 | 4 | 5 | 1 |
| Dust | غبار | Neutral | 4.491228 | 5 | 1.197062 | 5 | 5 | 0 |
| Vicious | شديد | Affect | 4.77193 | 5 | 0.567499 | 5 | 5 | 0 |
| Pressing | ضاغط | Sensory | 4.508772 | 5 | 0.947229 | 5 | 5 | 0 |
| House | منزل | Neutral | 4.596491 | 5 | 1.049824 | 5 | 5 | 0 |
| Killing | قاتل | Affect | 4.438596 | 5 | 1.295839 | 5 | 5 | 0 |

**Appendix 7: Demographic characteristics of the chronic pain group and healthy control group.**

| Demographic variable | | Chronic pain group (N=58) | Healthy Control group (N=57) | Group comparison |
| --- | --- | --- | --- | --- |
| Age | | M = 42.03, SD = 13.40 | M = 39.32, SD = 14.46 | W = 1887.5, p = 0.190 |
| Gender | Male | 60.35% (N=35) | 60% (N=34) | Matched |
|  | Female | 39.65% (N=23) | 40.35% (N=23) |  |
| Education | Primary level (equivalent to high school level UK) | 8.62% | 12.28% | X^2^ (3, N = 115) = 1.33, p = 0.722 |
|  | Secondary school level (equivalent to college level UK) | 29.31% | 22.81% |  |
|  | Undergraduate level | 44.83% | 42.11% |  |
|  | Postgraduate level | 17.24% | 22.81% |  |
| Income level | High | 5.17% | 5.26% | X^2^ (2, N = 115) = 2.13, p = 0.345 |
|  | Medium | 63.79% | 75.44% |  |
|  | Low | 31.03% | 19.30% |  |
| Marital status | Single | 17.24% | 47.37% | X^2^ (3, N = 115) = 13.85, p = 0.003 |
|  | Married | 72.41% | 50.88% |  |
|  | Widow | 8.62% | 1.75% |  |
|  | Divorced | 1.72% | 0.00% |  |
| Country of residence | Jordan | 56.9% (N=33) | 57.89% (N=33) | Matched |
|  | United Kingdom | 43.1% (N=25) | 42.11% (N=24) |  |

**Appendix 8: Means and standard deviations of the comorbid symptoms and pain characteristics variables.**

| Variable assessed | CP Group | Healthy Group | Groups comparison |
| --- | --- | --- | --- |
| Anxiety | *M* = 7.40,  *SD* = 5.05 | *M* = 5.54,  *SD* = 4.58 | *t*(113) = -2.07, *se* = 0.90, 95% *CI* [ -3.64, -0.08], *p* = .041 |
| Depression | *M* = 9.55,  *SD* = 5.61 | *M* = 7.33,  *SD* = 5.53 | *t*(113) = -2.14, *se* = 1.04, 95% *CI* [-4.28, -0.16], *p* = .035 |
| Pre-experiment Pain intensity | *M* = 5.83,  *SD* = 2.04 | *M* = 0.79,  *SD* = 1.37 | *t*(113) = -15.53, *se* = 0.33, 95% *CI* [-5.68, -4.40], *p <* .0001 |
| Post-experiment Pain intensity | *M* = 5.79,  *SD* = 2.03 | *M* = 0.76,  *SD* = 1.41 | *t*(113) = -15.41, *se* = 0.33, 95% *CI* [-5.68, -4.38], *p <* .0001 |
| Pain qualitative descriptive scale-pre experiment | *M* = 3.64,  *SD* = 1.02 | - | - |
| Pain qualitative descriptive scale-post experiment | *M* = 3.53,  *SD* = 1.17 | - | - |
| Duration of the pain (month) | M = 7.11,  SD = 6.41 | - | - |

**Appendix 9: Between groups t-test and within group paired t-test used for means comparison. Uncued minus cued reaction time; CP: chronic pain; HC: healthy control.**

| **Between groups (CP-HC)** | **t-value** | **DF** | **mean of difference** | **Standard error (se)** | **95% CI of the mean of the difference** | **P-value after Bonferroni correction** |
| --- | --- | --- | --- | --- | --- | --- |
| Sensory-Sensory | 1.36 | 112 | -0.028 | 0.021 | [-0.07, 0.013] | .178 |
| Affect-Affect | 1.79 | 111 | 0.036 | 0.02 | [-0.004, 0.076] | .076 |
| Neutral-Neutral | 2.9 | 109 | 0.052 | 0.022 | [0.009, 0.096] | .019 |
| **Within-group (CP)** | - | - | - | - | - | - |
| Sensory-Neutral | 2.44 | 112 | -0.054 | 0.022 | [-0.098, -0.01] | .016* |
| Affect-Neutral | 0.69 | 113 | -0.015 | 0.023 | [-0.061, 0.03] | .492 |
| Affect-Sensory | 1.86 | 113 | -0.039 | 0.021 | [-0.079, 0.003] | .066 |
| **Within-group (HC)** | - | - | - | - | - | - |
| Sensory-Neutral | 1.29 | 109 | 0.017 | 0.021 | [-0.014, 0.067] | .199 |
| Affect-Neutral | 0.04 | 107 | 0 | 0.019 | [-0.036, 0.037] | .927 |
| Affect-Sensory | 1.28 | 110 | 0.02 | 0.02 | [-0.01, 0.07] | .205 |

**Appendix 10: Violin plot of the spread of resilience total score for the chronic pain group (CPG) versus the healthy control group (HCG); the green spots colour grading indicates the density of participants scoring on the scale.**


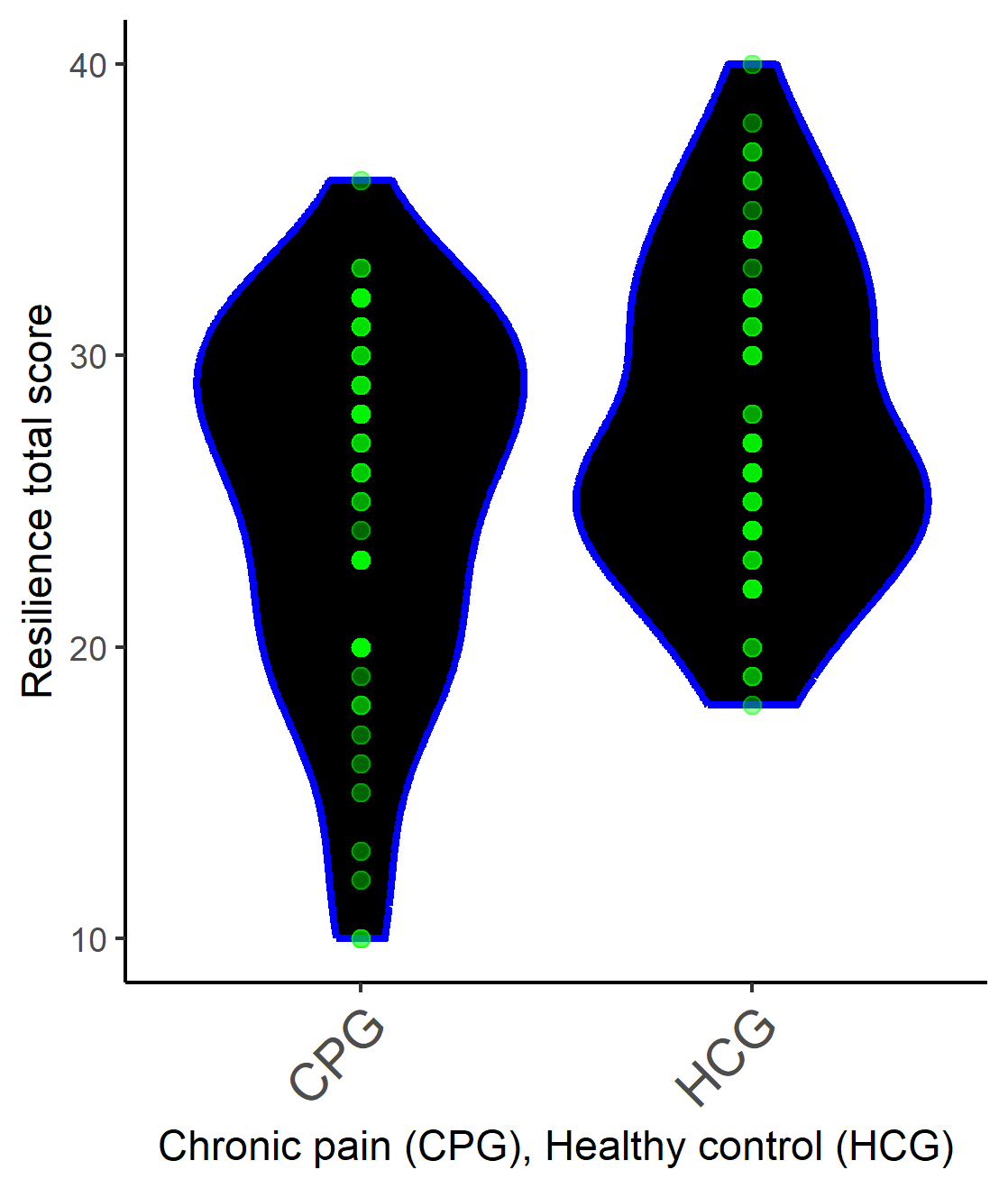


kk

**Appendix 11: Moderation effect of resilience on reaction time in Posner tasks for CP group (CPG) and healthy control group (HCG). The Y-axis shows the differences between the uncued minus cued effect, X-axis shows the resilience score for each participant. The lines represent the mean of the reaction time versus each resilience score mean for each word type (red = neutral words, green = affect words, and blue = sensory words).
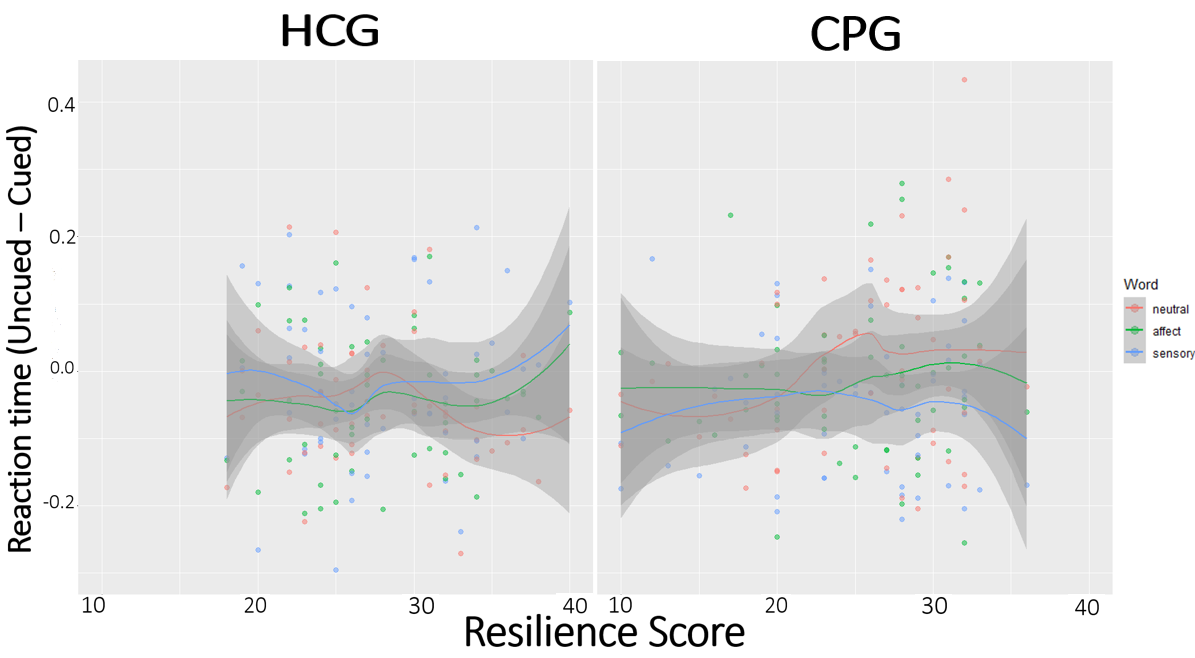
**
